# Cesarean section to prevent mother-to-child transmission of hepatitis B virus in China: A meta-analysis

**DOI:** 10.1186/s12884-017-1487-1

**Published:** 2017-09-12

**Authors:** Mei Yang, Qin Qin, Qiong Fang, Lixin Jiang, Shaofa Nie

**Affiliations:** 1 Futian District Center for Disease Control and Prevention of Shenzhen China, Futian District, Shenzhen, 518000 China; 2 Department of Health Prevention and Care, Zhongshan Hospital, Xiamen University, Xiamen, 361004 China; 3 Department of Epidemiology and Biostatistics, School of Public Health, Tongji Medical College, Huazhong University of Science and Technology, 13 Hangkong Road, Wuhan, 430030 China

**Keywords:** Cesarean section, Hepatitis B virus, Mother-to-child transmission, Meta-analysis

## Abstract

**Background:**

Hepatitis B virus (HBV) is predominantly transmitted through mother-to-child transmission (MTCT). To date, it remains unclear whether the method of parturition affects MTCT of HBV. In order to clarify whether cesarean section, when compared with vaginal delivery, could reduce the risk of MTCT of HBV in China, we conducted this meta-analysis.

**Methods:**

A systematic literature search was performed of the PubMed (Medline), Embase, ISI Web of Science, China Biological Medicine Database, China National Knowledge Infrastructure, and VIP Database for Chinese Technical Periodicals databases for articles written in English or Chinese through July 2015.The reference lists of relevant articles were also scrutinized for additional papers. Randomized controlled trials, cohort studies, or case-control studies investigating the effect of delivery mode on MTCT of HBV were included.

**Results:**

This analysis involved 28 articles containing 30 datasets. The data encompassed 9906 participants. The MTCT rate of HBV was 6.76% (670 of 9906) overall, with individual rates of 4.37% (223 of 5105) for mothers who underwent cesarean section and 9.31% (447 of 4801) for those who underwent vaginal delivery. The summary relative risk (RR) was 0.51 (95%CI: 0.44–0.60, *P* < 0.001), indicating a statistically significant decrease in HBV vertical transmission via cesarean section compared with vaginal delivery. The heterogeneity among studies was moderate with an *I* ^*2*^of29.3%.Publication bias was not detected by the Egger’s and Begg’s tests, and the funnel plot was symmetric. In the subgroup analyses, maternal hepatitis B e antigen status and follow-up time did not affect the significance of the results, but hepatitis B immune globulin (HBIG) administration to mother and infant did.

**Conclusions:**

Cesarean section could reduce the risk of MTCT of HBV in comparison to vaginal delivery in China. However, owing to several limitations of our meta-analysis, future well-designed randomized controlled trials with adequate statistical power, might be a more appropriate next step.

## Background

Hepatitis B is a potentially life-threatening liver infection caused by hepatitis B virus (HBV). It is a major global health problem. It can cause chronic infection and puts people at high risk of death from cirrhosis and liver cancer. According to the World Health Organization, an estimated 240 million people have chronic hepatitis B infection [1]. More than 780,000 people die every year due to complications of hepatitis B infection, including cirrhosis and liver cancer [2]. Hepatitis B prevalence is highest in sub-Saharan Africa and East Asia, where 5–10% of the adult population is chronically infected. The infected population of China accounts for one-third of the infected population worldwide [3].

HBV is predominantly transmitted through mother-to-child transmission (MTCT). In China, 30–50% of HBV carriers are infected through vertical transmission [3, 4]. Since the introduction of universal screening of pregnant women, combined with passive and active immunoprophylaxis, the MTCT rate of HBV has declined significantly. Passive immunoprophylaxis consists of the administration of hepatitis B immune globulin (HBIG) at birth, and active immunoprophylaxis is performed by the administration of the hepatitis B vaccine according to the standard 3-dose schedule. Although HBIG and vaccines given at birth are able to reduce transmission in most cases, in 10% to 20% of cases of infected mothers, transmission still occurs, especially from mothers with high viral loads and who are positive for hepatitis B e antigen (HBeAg) [5, 6].

Previous studies demonstrated that cesarean section might reduce the risk of MTCT for some viruses, such as human immunodeficiency virus and herpes simplex virus [7–9]. Numerous studies in China have sought to examine the relationship between delivery mode and rate of MTCT of HBV [10–16]. However, the effect of different delivery modes on MTCT of HBV remains unclear. To elucidate the question of whether cesarean section, as compared to vaginal delivery, may reduce the risk of MTCT of HBV, we conducted this meta-analysis.

## Methods

### Search strategy

A systematic literature search was performed of the PubMed (Medline), Embase, ISI Web of Science, China Biological Medicine Database, China National Knowledge Infrastructure, and VIP Database for ChineseTechnical Periodicals databases for articles written in English or Chinese through July 2015.The following search terms were used:“hepatitis B virus” or “HBV,”, “mother-to-child transmission” or “vertical transmission”, “cesarean section” or “vaginal delivery” or “natural delivery” or “delivery mode”. We also scrutinized the reference lists of relevant articles included in our analysis and previous systematic reviews and meta-analyses for additional papers. We did not contact the authors of the primary studies for additional information.

### Study selection

Studies were included in this meta-analysis if they satisfied the following criteria:

1) Studies were randomized controlled trials, cohort studies, or case-control studies; 2) the participants were pregnant mothers with chronic HBV infection, defined as maternal blood positive for HbsAg; 3) the intervention of interest was cesarean section and the comparison was vaginal delivery; 4) the outcome was vertical transmission of HBV, defined as infant HBsAg and/or HBV DNA positivity. Reviews, editorials, case reports/series, overlapping studies, and duplicates were excluded. We also excluded studies with patients co-infected with hepatitis C virus, hepatitis D virus, and/or human immunodeficiency virus. If the same population was studied in more than one study, we included the study with the largest sample size.

### Quality assessment

Our study used a 9-score system based on the Newcastle-Ottawa Scale (NOS) to assess the original study quality. The maximum score of 9 points could be assigned to a study that had the highest methodological quality, with 4, 2, 3 scores respectively being assigned to selection of study groups, comparability of study groups, assessment of outcomes, and adequacy of follow-up. Studies with scores of 0–3, 4–6, or 7–9 were regarded as low, moderate, and high quality, respectively (Table 1).

**Table 1:** Quality assessment of included studies

Studies	Selection				Comparability	Outcome			Total scores
	Representativeness of the exposed group	Selection of the unexposed group	Ascertainment of exposure	Outcome of interest not present at start of study	Control for important factor or additional factor	Outcome assessment	Follow-up long enough for outcome to occur	Adequacy of follow-up of cohorts	
Wang Qingtu, 2001 [45]	1	1	1	1	2	1	–	–	7
Wang Jianshe, 2002 [16]	1	1	1	1	2	1	1	1	9
Wang Lan, 2004 [44]	1	1	1	1	–	1	1	1	7
Wang Yuan, 2005 [43]	1	1	1	1	2	1	1	1	9
Gu Jie, 2006 [42]	1	1	1	1	2	1	1	1	9
Qian Yanhua, 2006 [41]	1	1	1	1	1	1	1	1	8
Fan Yi, 2007 [40]	1	1	1	1	2	1	1	1	9
Chen Jing1, 2007 [38]	1	1	1	1	1	1	1	1	8
Chen Jing2, 2007 [38]	1	1	1	1	1	1	1	1	8
Li Jijun1, 2007 [39]	1	1	1	1	1	1	1	1	8
Li Jijun2, 2007 [39]	1	1	1	1	1	1	1	1	8
Chen Hong, 2008 [37]	1	1	1	1	1	1	1	1	8
Liu Xia, 2008 [36]	1	1	1	1	–	1	1	1	7
Liu Honge, 2009 [35]	1	1	1	1	1	1	1	1	8
Zhang Qingying, 2009 [34]	1	1	1	1	2	1	1	1	9
Zhu Yunxia, 2010 [32]	1	1	1	1	2	1	1	1	9
Yang Peifang, 2010 [33]	1	1	1	1	2	1	1	1	9
Yang Xiaomei, 2011 [30]	1	1	1	1	2	1	1	1	9
Zhang Weili, 2011 [31]	1	1	1	1	1	1	1	1	8
Huang Liu, 2012 [29]	1	1	1	1	2	1	1	1	9
Guo Zhen, 2013 [15]	1	1	1	1	2	1	1	1	9
Wang Ling, 2013 [28]	1	1	1	1	2	1	1	1	9
Hu Yali, 2013 [14]	1	1	1	1	2	1	1	1	9
Yin Yuzhu, 2013 [12]	1	1	1	1	2	1	1	1	9
Pan Calvin Q., 2013 [13]	1	1	1	1	1	1	1	1	8
Zhang Lei, 2014 [11]	1	1	1	1	2	1	1	1	9
Wang Lina, 2014 [26]	1	1	1	1	2	1	1	1	9
Li Weiru, 2014 [25]	1	1	1	1	2	1	1		9
Huang Dan, 2014 [27]	1	1	1	1	2	1	1	1	9
Liu Cuiping, 2015 [10]	1	1	1	1	2	1	1	1	9

### Data extraction

Two authors (MY and QQ) independently conducted the data extraction process using a standard extraction form. When discrepancies arose, a third author (QF) was consulted. The following information was extracted from each study: first author’s name, publication year, study area, study design, number of infants born by cesarean section or by vaginal delivery, number of infants positive for HBV born by cesarean section or by vaginal delivery, percentage of mothers positive for HBeAg (HBeAg+), intervention administered (HBIG and/or antiviral treatment), percent age of infants who received HBIG, percent age of infants who received the HBV vaccine as scheduled, and the follow-up time of the study.

### Statistical analysis

In this meta-analysis, the relative risk (RR) and 95% confidence interval (CI) of HBV MTCT in cesarean section compared to vaginal delivery were considered as the effect size for all studies. Any results stratified by maternal HBeAg status were treated as two separate reports.

The heterogeneity between studies was assessed using the Cochran Q test and *I* ^*2*^ statistic [17]. For the *I* ^*2*^ metric, *I* ^*2*^ values of 25, 50, and 75% represent low, moderate, and high degrees of heterogeneity, respectively [18]. The fixed effect model (Mantel-Haenszel method) was applied to pool the effect when heterogeneity level was low; otherwise, the random effect model described by DerSimonian and Laird was utilized [19].

Subgroup analyses were conducted according to maternal HBeAg status, maternal HBIG administration and/or antiviral treatment, infant immunoprophylaxis status with HBIG, and follow-up time. Sensitivity analysis was performed by omitting one study at a time to examine the influence of each individual study on the overall relative risk. Publication bias was evaluated by Egger’s regression test [20], Begg’s adjusted rank correlation test [21], and visual inspection of the funnel plot. All tests were two-sided and *P* < 0.05 was considered statistically significant. All statistical analyses were conducted with Stata 11.0 (StataCorp, College Station, TX).

## Results

**Fig. 1:**
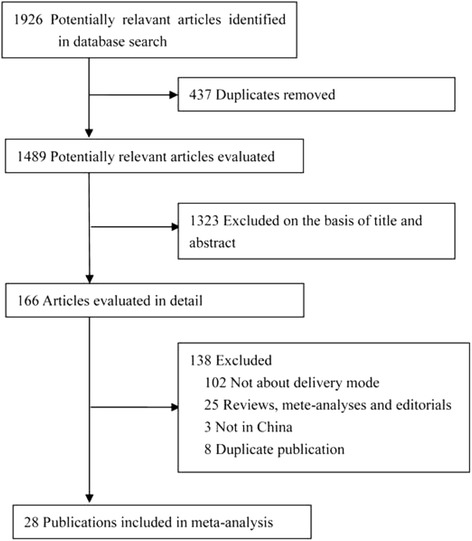
The flow diagram for literature research and study selection

The flow diagram for literature research and study selection is shown in Fig. 1. After screening the title and/or abstract of 1489 potentially relevant articles, 166 articles were evaluated in detail. Of these, 3 articles [22–24] were excluded for not having been conducted in China. Finally, 28 publications [10–16, 25–45] were eligible for inclusion in the meta-analysis.

Table 2 displays the main characteristics of the identified studies on the effect of delivery mode on MTCT of HBV. All included articles were published in the period between 2001 and 2015. Among the articles, 2 were case-control studies. All of the others were retrospective cohort studies. Seven were published in English, and the others were published in Chinese. Two articles reported the results grouped by maternal HBeAg status. Six studies were conducted exclusively in HBeAg + mothers, two were conducted solely in HBeAg-mothers, eight did not specify this information, and the remaining studies had mixed HBeAg + and HBeAg- populations (HBeAg + range from 3.83% to 67.19%). Two studies were conducted among pregnant women who all received HBIG administration, nine were conducted among those without HBIG administration, 15 did not specify, and four had mixed populations (HBIG range from 21.93 to 80%). One study was conducted with 7.81% of the mothers having received antiviral therapy, 14 were conducted among those who had not received antiviral therapy, and the remaining did not report whether antiviral therapy had been administered. Infants were tested for HBV within 24 h of birth before immunoprophylaxis in 6 studies. Among the studies with a follow-up period of more than 1 month, all infants received the hepatitis B vaccine in 24 studies. With regard to HBIG administration in infants in these 24 studies, 17 studies were conducted with 100% administration, 3 did not report this information, and 4 studies had a range from 53.3 to 84.34%.

**Table 2:** Characteristics of identified studies in this meta-analysis

			Study	Newborns		HBV+ newborns		Mothers		Infants		
Author	Year	Area	design	Caesarean	Vaginal	Caesarean	Vaginal	HBeAg + (%)	Intervention	HBIG	Vaccine	Follow-up
Wang Qingtu	2001	Shandong	RC	38	84	4	28	100	NR	NR	NR	1 h
Wang Jianshe	2002	Shanghai	RC	117	184	13	17	36.54	NR	100%	1, 2 and 7 months	12 months
Wang Lan	2004	Shandong	RC	61	183	11	61	100	NR	75.82%	0, 1 and 6 months	1 month
Wang Yuan	2005	Jiangsu	RC	58	42	1	0	22	100% HBIG	100%	0, 1 and 6 months	2 month
Gu Jie	2006	Ningxia	RC	64	85	2	5	34.9	No	100%	0, 1 and 6 months	12 months
Qian Yanhua	2006	Jiangsu	RC	26	24	0	2	NR	80% HBIG	80%	0, 1 and 6 months	12 months
Fan Yi	2007	Guangdong	RC	92	103	2	9	4.1	NR	100%	0, 1 and 6 months	6 months
Chen Jing1	2007	Tianjin	RC	176	53	27	13	100	NR	NR	NR	1 h
Chen Jing2	2007	Tianjin	RC	112	68	3	10	0	NR	NR	NR	1 h
Li Jijun1	2007	Gansu, Jiangsu	RC	12	49	4	19	100	NR	NR	0, 1 and 6 months	3–9 years
Li Jijun2	2007	Gansu, Jiangsu	RC	74	184	5	28	0	NR	NR	0, 1 and 6 months	3–9 years
Chen Hong	2008	Beijing	RC	112	180	4	5	4.45	No	NR	0, 1 and 6 months	6 months
Liu Xia	2008	Tianjin	RC	153	54	14	15	NR	NR	NR	NR	2 h
Liu Honge	2009	Guangdong	RC	86	123	2	9	3.83	NR	100%	0, 1 and 6 months	1 month
Zhang Qingying	2009	Shanghai	RC	166	52	1	7	NR	No	100%	0, 1 and 6 months	6 months
Zhu Yunxia	2010	Beijing	RC	114	102	5	6	100	No	100%	0, 1 and 6 months	7 months
Yang Peifang	2010	Shanghai	RC	32	36	0	1	19.1	No	100%	0, 1 and 6 months	12 months
Yang Xiaomei	2011	Guangdong	RC	33	49	4	7	NR	NR	100%	0, 1 and 6 months	6 months
Zhang Weili	2011	Shanghai	RC	508	127	17	5	NR	NR	100%	0, 1 and 6 months	3 months
Huang Liu	2012	Guangdong	RC	122	168	9	17	NR	NR	NR	NR	24 h
Guo Zhen	2013	Shanxi	CC	584	549	29	72	36.19	NR	NR	NR	24 h
Wang Ling	2013	Sichuan	RC	112	148	7	16	NR	100% HBIG	100%	0, 1 and 6 months	6 months
Hu Yali	2013	Jiangsu	RC	285	261	7	6	24.73	NR	53.30%	0, 1 and 6 months	5–7 years
Yin Yuzhu	2013	Guangdong	RC	692	668	12	9	34.76	39.70% HBIG	100%	0, 1 and 6 months	12 months
Pan Calvin Q.	2013	Beijing	RC	496	673	7	23	54.5	No	100%	0, 1 and 6 months	7–12 months
Zhang Lei	2014	Multicenter	RC	221	194	17	22	100	21.93% HBIG	84.34%	0, 1 and 6 months	8-12 months
Wang Lina	2014	Guangdong	RC	149	122	6	9	34.32	No	100%	0, 1 and 6 months	7–12 months
Li Weiru	2014	Guangdong	RC	120	120	2	12	NR	No	100%	0, 1 and 6 months	6 months
Huang Dan	2014	Hubei	RC	86	64	4	8	18.67	No	100%	0, 1 and 6 months	6 months
Liu Cuiping	2015	Sichuan	CC	204	52	4	6	67.19	7.81% antiviral; 43.36% HBIG	100%	0, 1 and 6 months	6–12 months and/or 1–3 years

### Meta-analysis

**Fig. 2:**
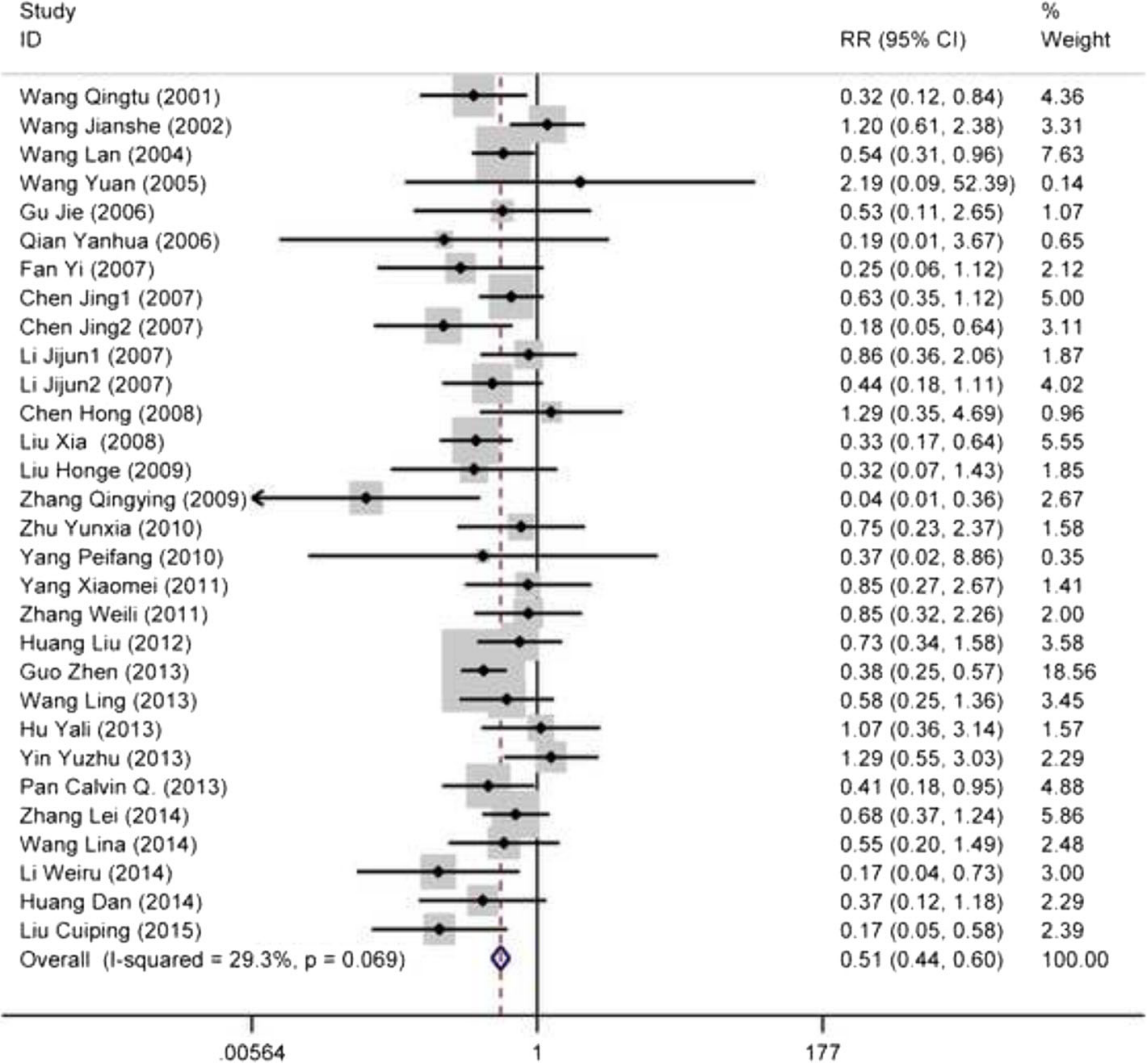
The forest plot of the efficacy of caesarean section versus vaginal delivery for preventing hepatitis B virus vertical transmission

**Fig. 3:**
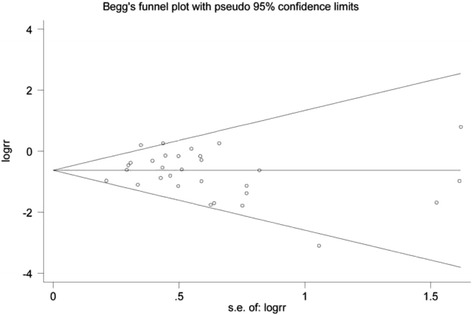
Funnel plot of publication bias

This analysis involved 28 articles with 30 datasets comprising 9906 participants. The MTCT rate of HBV was 6.76% (670 of 9906) overall, with individual rates of 4.37% (223 of 5105) for mothers who underwent cesarean section and 9.31% (447 of 4801) for those who underwent vaginal delivery. The study-specific RRs and the pooled estimate are shown in Fig. 2. The summary RR was 0.51 (95%CI: 0.44–0.60, *P* < 0.001), indicating a statistically significant decrease in HBV vertical transmission with cesarean section, as compared to vaginal delivery. The heterogeneity among studies was moderate with *I* ^*2*^of29.3% (*P*_*heterogeneity*_ = 0.069). In the sensitivity analysis, the pooled RRs were similar before and after removal of each study, indicating the robust stability of the current result. Publication bias was not detected by Egger’s (*P* = 0.452) and Begg’s (*P* = 0.254) tests, and the funnel plot was symmetric (Fig. 3).

Subgroup analyses were conducted to examine the stability of the primary result (Table 3). Significant results did not differ substantially by maternal HBeAg status and follow-up time. As for the stratified analyses by maternal HBIG administration, the risk of MTCT of HBV was significantly decreased in the cases of cesarean section when compared to vaginal delivery among pregnant women without HBIG administration and those where HBIG administration had not been specified, but not among those with HBIG administration (100% or mixed). A significant effect of cesarean section on the MTCT of HBV was found in those studies where either 100% or a smaller percentage of infants had been administered HBIG, as well as in studies where administration of HBIG to infants had not been reported. With regard to infant vaccination, the significant effect of cesarean section did not differ among infants who received vaccination and those whose vaccination status was unknown.

**Table 3:** Subgroup analyses on the effect of delivery mode on the risk of hepatitis B virus vertical transmission

Characteristic	No. of reports	RR (95% CI)	*P* for test	*I* ^*2*^(%)	*P* for heterogeneity
All studies	30	0.51 (0.44–0.60)	<0.001	29.3	0.069
Maternal HbeAg status					
HBeAg+	6	0.59 (0.44–0.78)	<0.001	0.0	0.731
HBeAg-	2	0.33 (0.16–0.68)	0.003	21.1	0.260
Mixed	14	0.53 (0.42–0.68)	<0.001	40.3	0.059
Not reported	8	0.45 (0.32–0.63)	<0.001	43.2	0.090
Maternal HBIG administration					
Yes (100%)	2	0.63 (0.28–1.44)	0.276	0.0	0.427
No	9	0.44 (0.27–0.72)	0.001	22.1	0.247
Mixed	4	0.54 (0.23–1.30)	0.169	61.2	0.052
Not reported	15	0.54 (0.42–0.70)	<0.001	32.9	0.105
Infant HBIG administration					
Yes (100%)	17	0.55 (0.42–0.71)	<0.001	36.9	0.064
Mixed	4	0.63 (0.43–0.92)	0.017	0.0	0.594
Not reported	9	0.45 (0.35–0.57)	<0.001	29.7	0.182
Infant vaccine					
0–1-6 months	23	0.55 (0.44–0.67)	<0.001	14.1	0.268
1–2-7 months	1	–	–	–	–
Not reported	6	0.41 (0.32–0.54)	<0.001	23.7	0.256
Follow-up time					
≥ 1 month	24	0.58 (0.48–0.71)	<0.001	22.7	0.157
≤ 24 h	6	0.41 (0.32–0.54)	<0.001	23.7	0.256
Region					
EDA	22	0.55 (0.45–0.67)	<0.001	34.8	0.056
CLDA	2	0.38 (0.26–0.56)	<0.001	0.0	0.978
WUDA	3	0.43 (0.23–0.80)	0.008	26.6	0.256
Mixed	3	0.63 (0.40–0.97)	0.037	0.0	0.573

Abbreviations: *HBeAg* hepatitis B e antigen, *HBIG* hepatitis B immune globulin, *RR* relative risk, *CI* confidence interval, *EDA* eastern developed areas, *CLDA* central less developed areas, *WUDA* western or undeveloped areas

## Discussion

In this meta-analysis, we found that cesarean section could significantly reduce the risk of MTCT of HBV in China. The significant effect was not affected by maternal HBeAg status and follow-up time, but was influenced by both maternal and infant HBIG administration. Our results are consistent with the findings of two previous meta-analyses [46, 47]; however, one only included 4 studies and another included 10 studies worldwide. In the meta-analysis reported by Chang et al. [46], most of the included studies were performed in China, but some Chinese articles were excluded owing to the lack of available translations. We were able to take full advantage of Chinese databases and include 28 studies performed in China in this meta-analysis, making it the largest and most robust meta-analysis to date.

The mechanism of MTCT of HBV remains unclear. Most MTCTs likely occur perinatally by microperfusion of maternal blood to the fetal circulation during the uterine contractions and tearing of the placenta at birth. Other possible modes of infection include swallowing amniotic fluid, vaginal secretions, or exposure to maternal blood during vaginal delivery [13]. Elective cesarean section results in the least amount of placental contraction and has been speculated to involve the least maternal-fetal transfusion [15]. It also limits direct contact of the fetus with infected secretions or blood in the maternal genital tract.

Several limitations of our meta-analysis should be acknowledged. Despite demonstrating the efficacy of cesarean section for preventing MTCT of HBV, the conclusion of this study must be considered with great caution. Firstly, the included studies were retrospective cohort and case-control studies, not randomized controlled trials. Some bias and confounding factors could not be well controlled in the retrospective studies. Secondly, the data on maternal HBV DNA level in the included studies were lacking, making it difficult to reach conclusions. The maternal HBV DNA level was considered as the most important independent risk factor for vertical transmission by many studies [5, 48, 49]. Thirdly, although we found the significant effect did not differ by maternal HBeAg status, in most of the studies we included, the authors did not report maternal HBeAg status, or the participants had mixed HBeAg statuses. HBeAg positivity had a close relationship with HBV DNA level. Finally, HBIG administration and antiviral therapy in pregnant women were not reported in most studies. The results may have been affected by the lack of these data. In addition, owing to missing clinical details on hepatitis B, varying study quality, and study heterogeneity, it would be difficult to justify a randomized trial of cesarean section, which is currently an unproven and invasive intervention. A detailed, well-designed observational study, where all infants receive HBIG and vaccination and all mothers experience perinatal hepatitis B care, might be a more appropriate next step.

## Conclusions

Our meta-analysis found that cesarean section could reduce the risk of MTCT of HBV in comparison to vaginal delivery in China. Whether elective cesarean section can be recommended for clinical practice as a preventive measure against MTCT of HBV should be considered with caution. Future well-designed randomized control trials with adequate statistical power will be needed to confirm our findings.

## Abbreviations

CLDA: Central less developed areas
EDA: Eastern developed areas
HBeAg: Hepatitis B e antigen
HBIG: Hepatitis B immune globulin
HBV: Hepatitis B virus
MTCT: Mother-to-child transmission
NOS: Newcastle-Ottawa Scale
WUDA: Western or undeveloped areas
